# Learning From the Past to Advance the Future: The Adaptation and Resilience of NASA’s Spaceflight Multiteam Systems Across Four Eras of Spaceflight

**DOI:** 10.3389/fpsyg.2019.01633

**Published:** 2019-07-12

**Authors:** Jacob G. Pendergraft, Dorothy R. Carter, Sarena Tseng, Lauren B. Landon, Kelley J. Slack, Marissa L. Shuffler

**Affiliations:** ^1^Department of Psychology, University of Georgia, Athens, GA, United States; ^2^KBRwyle, Houston, TX, United States; ^3^National Aeronautics and Space Administration, Washington, DC, United States; ^4^Department of Psychology, Clemson University, Clemson, SC, United States

**Keywords:** teams, multiteam systems, spaceflight, adaptive performance, organizational practices, evolution and adaptability

## Abstract

Many important “grand” challenges—such as sending a team of humans on a voyage to Mars—present superordinate goals that require coordinated efforts across “multiteam systems” comprised of multiple uniquely specialized and interdependent component teams. Given their flexibility and resource capacity, multiteam system structures have great potential to perform *adaptively* in dynamic contexts. However, these systems may fail to achieve their superordinate goals if constituent members or teams do not adapt their collaboration processes to meet the needs of the changing environment. In this case study of the National Aeronautics and Space Administration (NASA)’s *Spaceflight Multiteam Systems* (SFMTSs), we aim to support the next era of human spaceflight by considering how the *history* of manned spaceflight might impact a SFMTS’s ability to respond adaptively to future challenges. We leverage archival documents, including Oral History interviews with NASA personnel, in order to uncover the key attributes and structural features of NASA’s SFMTSs as well as the major goals, critical events, and challenges they have faced over 60 years of operation. The documents reveal three distinct “eras” of spaceflight: (1) *Early Exploration*, (2) *Experimentation*, and (3) *Habitation*, each of which reflected distinct goals, critical events, and challenges. Moreover, we find that within each era, SFMTSs addressed new challenges adaptively by modifying their: (1) technical capabilities; (2) internal collaborative relationships; and/or (3) external partnerships. However, the systems were sometimes slow to implement needed adaptations, and changes were often spurred by initial performance failures. Implications for supporting future SFMTS performance and future directions for MTS theory and research are discussed.

## Introduction

The United States’ National Aeronautics and Space Administration (NASA) and directives from the President have set an ambitious goal: send manned Long-Duration Exploration Missions (LDEMs) to deep-space destinations like Mars within the next two decades (National Aeronautics and Space Administration [NASA], 2014; Trump, 2017). LDEMs represent a new frontier for humanity, and could be one of the greatest achievements in human history. However, these missions will also present immense difficulties and test the capabilities of all involved. Factoring prominently among the anticipated difficulties of LDEMs is the *team risk* or the “risk of performance and behavioral health decrements due to inadequate cooperation, coordination, communication, and psychosocial adaptation within a team” (Landon et al., 2016, p. 5). The “team risk” in a LDEM is not limited to the risks of collaboration failures within the spaceflight *crew*. LDEMs will require unprecedented levels of collaboration across complex *“spaceflight multiteam systems”* (i.e., “SFMTSs”) comprised of the space flight crew and numerous teams on Earth (Mesmer-Magnus et al., 2016).

In fact, *many* of the most important problems facing today’s organizations and societies —including responding to natural disasters (DeChurch et al., 2011), uncovering major scientific discoveries (Falk-Krzesinski et al., 2010), and translating medical breakthroughs to practice (Asencio et al., 2012)—represent “grand challenges” (George et al., 2016) that require intensive collaboration across interdependent systems comprised of multiple uniquely specialized groups or teams. These *“teams of teams”* or *“multiteam systems”* (i.e., “MTSs”; Mathieu et al., 2001) are increasingly prevalent in today’s world because these structures offer greater resource capacity than single teams but more flexibility than traditional organizations and thus, are expected to respond *adaptively* to complex and evolving task demands (Marks et al., 2005; Porck et al., 2018).

Despite their potential to achieve important goals, extant research suggests that MTSs often fail due to breakdowns in collaboration and coordination within and/or across component teams (Zaccaro et al., 2012). For example, MTS theory argues that *interteam* collaboration breakdowns are particularly likely in systems comprised of teams with very different areas of expertise, backgrounds, norms, priorities, or organizational memberships (Luciano et al., 2018). Furthermore, MTSs often appear in contexts that are ambiguous, dynamic, multi-faceted, and require rapid responses (Shuffler and Carter, 2018). Yet, research on dynamic task contexts suggests that dynamism and uncertainty can present added problems for collaboration (Luciano et al., 2018) and members and teams may fail to shift their processes and procedures adaptively to meet evolving task demands (Moon et al., 2004; Hollenbeck et al., 2011). Therefore, when MTSs face an important grand challenge, like a LDEM, which has critical consequences for failure, it is often necessary to understand the specific features of the system (e.g., team characteristics, evolving task demands) that might present barriers to effective collaboration within and across teams and develop strategies for mitigating those barriers.

This case study aims to lay a foundation for supporting SFMTS performance in the future by analyzing the *history* of SFMTS performance over the past 60 years of NASA’s spaceflight program. We argue that considering the collaboration practices and procedures that have been established *previously* within a MTS or its embedding environment is an important first step when attempting to facilitate future adaptive performance. Indeed, scholars have long argued that teams’ histories can substantially impact their futures (McGrath et al., 2000; Hollenbeck et al., 2014). Through a review of archival documents, we uncover the key features of SFMTSs and the major focuses, critical events, and challenges SFMTSs have contended with in the past. Further, we consider the ways in which SFMTSs have *adapted* to meet the challenges of previous eras of spaceflight. In doing so, we align with previous research on teams that acknowledges “adaptation lies at the heart of team effectiveness” (Burke et al., 2006, p. 1189) and identify aspects of prior adaptations within the spaceflight context that must shift or advance further in order to achieve the goals of LDEM.

## Case Study Approach

The purpose of this research is to better understand how NASA’s SFMTSs have learned from and adapted in response to pivotal events and transitions in the space program over the past 60 years of space exploration. Toward these ends, we reviewed publicly available archival documents that provide first-hand information regarding how NASA’s SFMTSs responded to critical events. Our case study was guided by three research questions which were grounded in extant theory and research on MTSs (Zaccaro et al., 2012; Shuffler et al., 2015). These research questions, our data collection, and analysis procedures are described below.

### Research Questions

#### Research Question 1

Our first research question *How are NASA’s SFMTSs structured?* (e.g., What teams are involved? What interteam relationships are relevant?) is based in prior theoretical work which has identified the key definitional features of MTSs (Mathieu, 2012) and delineated the attributes of these systems that might impact performance (Zaccaro et al., 2012). Defined formally, MTSs are: “two or more teams that interface directly and interdependently in response to environmental contingencies toward the accomplishment of collective goals” (Mathieu et al., 2001, p. 289). All MTSs have in common two features: two or more component teams, and a hierarchical goal structure whereby component team pursue separate team-level goals in addition to one or more shared “superordinate” goal.

However, as Zaccaro et al. (2012) argue MTSs can vary widely with regard to the types of “compositional,” “linkage,” and “developmental” attributes affecting MTS functioning. *Compositional attributes* are descriptive aspects of the individuals and teams comprising the system and can include demographic features of the MTS, the size of the system (e.g., number of teams), the relative characteristics of the component teams (e.g., the functional specialization of component teams), and the degree to which the system crosses organizational boundaries. *Linkage attributes* reflect the formal and informal connections among members and teams and can include patterns of task interdependence driven by the MTS goal hierarchy, communication, trust, and leadership structures. Finally, *developmental attributes* are the properties of the system connected to temporal development such as the system’s genesis (e.g., if the system was appointed or emergent), and the stability of the membership over time.

As a guiding theoretical framework, MTSs researchers typically leverage classic *input-process-output* (Steiner, 1972; McGrath, 1984; Hackman, 1987) or *input-mediator-output-input* (IMOI model; Ilgen et al., 2005) views of team functioning and performance to understand multiteam functioning. Within these models, inputs reflect factors affecting team functioning (e.g., personality, knowledge, training, attitudes). The effects of inputs are transmitted through mediators, such as teamwork processes (e.g., coordination behaviors, information sharing, backup behaviors; Marks et al., 2001) or emergent psychological states (e.g., trust, shared cognition; Kozlowski and Ilgen, 2006) to team outputs (e.g., performance, viability). In MTSs, inputs (e.g., compositional attributes; Zaccaro et al., 2012) residing at the individual, component team, and system level shape the interactions and relationships within and across teams (e.g., linkage attributes), and MTS outcomes. These performance outcomes then become inputs during subsequent phases of performance.

In summary, extant research argues that MTSs can vary widely in their structures and other compositional, linkage, and developmental attributes. Moreover, the structures and attributes of MTSs are significant determinants of systems performance. For example, drawing from a long history of research on intergroup relations (Sherif, 1958; Tajfel et al., 1979), Luciano et al. (2018) argue that the degree to which component teams differ from one another with regard to their functional capabilities, norms, work processes, and priorities, can create boundary-enhancing forces between teams that stifle interteam collaboration and system performance. Therefore, our first research question is based in the understanding that MTS structures and other attributes are critical to system performance.

#### Research Questions 2 and 3

Although research on organizational teams has often treated team tasks, composition, and environments as though they were stable over time (Ilgen, 1999; Mathieu et al., 2017), scholars have also pointed out that teams and MTSs are complex adaptive systems that experience evolving task demands, shifting group memberships, and feedback loops with their embedding environments (Kozlowski and Klein, 2000; McGrath et al., 2000; Mathieu et al., 2014). The prior experiences, outcomes, memories, and practices that have accumulated within a team or system in response to evolving task demands are likely to shape subsequent behaviors and outcomes (e.g., McGrath et al., 2000; Hollenbeck et al., 2014). Moreover, a team or system’s ability to *adapt* to major changes is a hallmark of effective performance (LePine, 2005; Burke et al., 2006; Baard et al., 2014). Therefore, the second two research questions guiding our case study of NASA’s SFMTSs acknowledge that teams’ *histories* (and their prior adaptations) matter to their futures: (2) *What are major goals, critical events, and challenges have NASA’s SFMTSs faced in the past?*; and (3) *In what ways have NASA’s SFMTSs adapted over time in response to evolving goals, events, and challenges?* (e.g., What organizational practices have been implemented?).

The history of a MTS might facilitate subsequent performance or constrain it. In some instances, when future challenges share similar features to those encountered in the past, prior adaptations represent a valuable resource which teams may draw on to inform their options for future adaptation. Where anticipated challenges diverge from those encountered previously, a thorough understanding of past challenges and the adaptations made in response to them may guide subsequent adaptation strategies by allowing team members to identify the areas where further improvement on existing systems may be needed. Conversely, circumstances may require teams to change their behaviors, but reliance on past approaches may prevent adaptation. For example, research has shown that it is much easier for teams to shift from loosely coupled or decentralized task decision-making structures toward more tightly coupled or centralized structures than it is to shift in the opposite direction (Moon et al., 2004; Hollenbeck et al., 2011).

Therefore, we consider the ways in which NASA’s SFMTSs have previously adapted to evolving challenges. We suggest that considering the history of SFMTS adaptations could provide a foundation for future LDEMs. First, an awareness of past adaptations may provide guidelines for the types of adaptations that may benefit the system in the future. Second, understanding prior challenges may allow for better prediction of the performance decrements that may result from the challenges of LDEMs if further adaptations are not instituted. Finally, an advance awareness of potential performance decrements may allow NASA and organizational researchers to apply countermeasures, correcting for these challenges before their consequences can manifest. Examining the past to inform the future may be particularly important in multiteam settings like an SFMTS, which could differ appreciably from less complex stand-alone teams studied in laboratory settings or other types of organizations.

### Data Collection Approach

We used transcripts from NASA’s JSC Oral History Project (JSC OHP) as the foundation of our archival document search. The purpose of the JSC OHP was to “capture the history from the individuals who first provided the country and the world with an avenue to space and the moon” (Madison, 2010). The JSC OHP transcripts represent interviews with individuals spanning a wide range of roles within NASA, including managers, engineers, technicians, astronauts, and other employees. Our review was conducted entirely using publicly available documents. As such, additional IRB, NASA, or interview participant approval was not required for the use of these resources.

We used the JSC OHP as the foundation of our archival analysis for three key reasons. First, by virtue of their inclusion in the JSC OHP, the events described in the transcripts can be assumed to be of importance to the organization, from the perspective of NASA itself. These events often represented critical milestones in NASA’s spaceflight legacy. In many cases, this was because the events described were pivotal in prompting altered patterns of action that were key to later successes, or marked the surmounting of persistent and lasting problems which would establish a template for future action. Often, the focus of the interviews could be described as “crisis” events, although significant successes were also frequent topics. Therefore, although the documents largely exclude day-to-day functioning of NASA and MCC which is sure to have substantial impacts on the operation of the system as well, the OHP provides an ideal basis for identifying pivotal events and transitions within the space program. Although the events that are the focus of the JSC OHP represent a small proportion of the totality of NASA’s 60-year history, these events continue to exercise disproportionate impact on NASA’s operations.

Second, the JSC OHP documents represented first-hand accounts of pivotal events and NASA transitions from the perspective of interview subjects who were intimately familiar with and/or played a prominent role in the events described. The selection of oral history project subjects was often guided by the familiarity of the subject with one or more formative events or periods in the history of the organization. The interview transcripts are presented with limited revisions to preserve their conversational tone, and typically range between approximately 30 and 60 pages per interview. Participants were prompted by a NASA oral historian—whose questions are recorded in the transcripts—to recall their personal experiences and perceptions of prominent events or periods in NASA’s history.

Third, the subjects of the oral histories tended to provide a substantial amount of detail in terms of the intrapersonal states (e.g., stress levels, motivation, affect, etc.) and interpersonal relationships and behaviors (e.g., trust, shared cognition, information sharing) acting on the system at the time of the events in question. Details about internal states and interpersonal relationships and motivational factors are frequently omitted from more formal technical records but are highly relevant to the functioning of MTSs (Zaccaro et al., 2012; Rico et al., 2017; Luciano et al., 2018). The type of unique insights into the internal and interpersonal states gleaned through the JSC OHP documentation are exemplified by the following quote from astronaut Michael Foale, regarding the aftermath of the collision of an unmanned *Progress* resupply spacecraft with the *Mir* station:

*“So that was a pretty hard time, because we got very tired. And that was the hardest time I ever had on the station, was that period, because we just got so tired. Of course, the commander’s morale was pretty – he was just shot, stunned.”* – Foale (1998, 16 June), astronaut.

#### Collection of Archival Documents

Our collection of archival documents progressed in a series of three steps and leveraged an adapted snowballing review technique (Wohlin, 2014). In the first step, we began by compiling all available transcripts from the JSC OHP (*n* = 374 transcripts). Then, the first and third authors read through each transcript and removed all transcripts that did not contain references to one or more *manned* space mission and/or did not make multiteam interactions a central focus of the interview. This resulted in a much smaller subset of 30 focal JSC OHP transcripts containing information relevant to our research questions. These sources explicitly discussed SFMTS collaboration during a manned space mission. The decision to focus on multiteam collaboration involving members of NASA’s MCC, along with our restricted focus on manned spaceflight missions, was guided by the recognition that “crew-ground” relations—between members of the spaceflight crew and MCC personnel—will be critical to the success of future space exploration missions to deep space destinations (Landon et al., 2018).

In many cases, the JSC OHP interviewees referenced events and mission details but did not explain the technical details of the events and/or the longer-term decisions that were made in response to the events thoroughly. For example, the following quote from an oral history interview with NASA flight engineer Christopher Kraft regarding the early stages of the Spacelab program demonstrates the type of statement which required more explanation:

*“It just was sort of a long arduous task to get anything done…You know what the arrangement was.”* – Kraft (1991, 28 June), Flight Engineer (underlined emphasis added).

Therefore, in the second step of our data collection, we generated a list of all of the manned spaceflight missions referenced in the 30 focal JSC OHP transcripts. Then, we gathered official NASA- or government agency-produced documentation (e.g., investigation reports, government announcements, international agreements, etc.) related to the focal events in order to supplement our understanding of these events (*n* = 18 official documents). In cases where these documents also lacked sufficient detail, we gathered additional sources (*n* = 60 additional sources) that provided more detail about the events in question. These additional sources included NASA articles (e.g., online blogs), mission archives (i.e., overview descriptions of mission goals, technical aspects, and task focus), other NASA documents (e.g., NASA history office gallery entries), and articles from external news sources. The additional NASA documentation was instrumental in helping us establish a clearer view of the situational facts of many events, particularly the granular details of individual missions. In total, these first two data collection steps resulted in a total of 108 sources.

**FIGURE 1:**
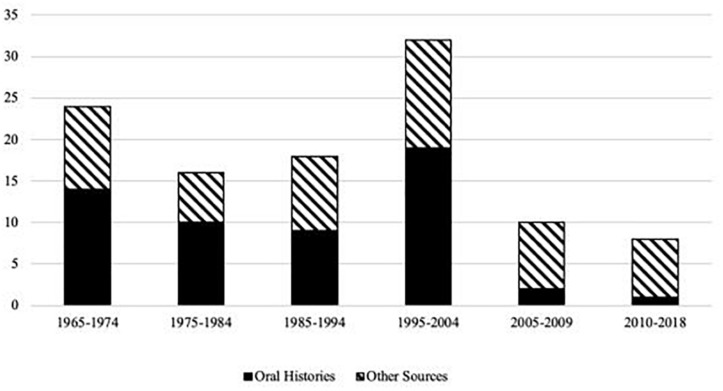
Frequency of critical events across decades and source type.

In a third step, two Subject Matter Experts (SMEs), who are intimately familiar with the history of NASA, refined the initial set sources by eliminating sources which referenced events the SMEs did not believe had played a significant role in the history of the organization and/or any sources that they deemed to be unreliable or inaccurate. Specifically, the majority of excluded documents were removed due to their irrelevance to central developments in the history of NASA (*n* = 22), while a smaller proportion were removed due to inaccuracies or inconsistencies (*n* = 6). The majority of these six cases were excluded due to inconsistencies with other NASA documentation regarding the chief causes of events, as well as factual inconsistencies identified by comparison with other sources in a minority of cases. This SME evaluation process resulted in a final set of 80 sources. Appendix A provides a complete list of these sources. These sources discussed events occurring between 1960 and the present day, roughly spanning the operational history of NASA’s MCC. Table 1 and Figure 1 summarize the types of resources identified and their frequencies by year, respectively.

**TABLE 1:** Summary of resources included in archival analyses.

**Source Type**	**Count**
NASA Oral Histories	30
Official NASA or government reports	11
NASA articles, NASA mission archives, other NASA documents, articles from external news outlets	39

### Analysis of Archival Documents

Our research team coded each of the events described in the identified sources in order to identify the answers to our three research questions. To begin, the first three co-authors read each of the sources and generated answers to the research questions independently. Then, the coding team met and came to a group consensus regarding the answers to the three research questions. Lastly, the coding team’s findings were then evaluated and refined by two SMEs familiar with the functioning and history of NASA.

Answers to the research questions were primarily derived from the oral history interview documents and were extracted for each of the focal events. For example, information about the structure of the system and the nature of the component teams was frequently available from the oral histories themselves as was a great deal of information pertaining to the interteam relationships within the system. The following quote from William Reeves exemplifies this:

*“They assigned me to head up the first consultant group that went over to Russia, to their Control Center, to support from their Control Center, real time. At the same time, there was a group of Russians that came over here, Russian flight controllers, that formed a consultant group that was in our Control Center.”* – Reeves (1998, 22 June), flight controller.

Likewise, the goals and challenges of relevant missions were frequently discussed by the interviewees, who were typically acutely aware of them. For example, Michael Barratt responds to a prompt to discuss challenges early in an interview:

*“I think some of the most significant challenges, of course, were working with our international partners. In particular working with our former Cold War adversaries, our Russian friends.”* – Barratt (2015, 30 July), flight surgeon and medical systems designer.

When additional information on mission goals was required, the supplemental documents (e.g., mission logs) frequently provided sufficient detail through stated mission objectives. System adaptations were frequently described in the oral histories as well, although these also tended to appear in more explicit detail in investigation reports following performance failures. For example, the *Report of the Presidential Commission on the Space Shuttle Challenger Accident* contains sections explicitly detailing the actions taken to implement the recommendations of the commission (Presidential Commission on the Space Shuttle Challenger Accident, 1986). Throughout, where quoted material appears in the text, bracketed material represents sparingly added text to provide clarity (drawing from statements elsewhere in the interview) and allow for concise quotation. Ellipses represent omitted text from the original statement, similarly used to limit the quotation to the required information.

## Case Study Findings: SFMTS Structures, Challenges, and Adaptations

### Research Question 1: How Are NASA’s SFMTSs Structured?

To Research Question 1, we evaluated the MTS structures in use during the manned spaceflight missions discussed in the JSC OHP transcripts and the relationships within and across teams that appear to be pivotal to SFMTS success. Prior work has identified the spaceflight crew and the teams comprising NASA’s Mission Control Center (MCC) as key component teams in a SFMTS and argued that *ground-crew* relations are critical to spaceflight mission performance (Landon et al., 2018). Located at Johnson Space Center (JSC) in Houston, Texas, United States, NASA’s MCC is the organization primarily responsible for directing a space exploration mission and monitoring the vehicle during manned space missions. The staff of MCC is chiefly tasked with ensuring the safety of the crew and the completion of mission objectives. Indeed, we identified many references to ground-crew relations in the archival documents. For example, astronaut Bonnie Dunbar discussed communication regarding various systems:

*“We had a Mission Control Center for the payloads in southern Germany, so that’s where we talked… to their engineers when we were operating the payloads, or we would talk to their researchers if they were enabled. If we wanted to talk about Spacelab systems, then we’d talk back to Houston… and so I would talk to both Houston and to* München.*”* — Dunbar (2005, 20 January), astronaut.

Interestingly, we also identified multiple references to ground-*ground* relations between members of distinct but interdependent component teams on Earth—particularly between front room and *backroom* teams in the MCC. For example, another quote from astronaut Bonnie Dunbar illustrates the importance of ground-ground relations to the success of the Shuttle-*Mir* program and the subsequent ISS:

*“I think flight crews are probably the easiest to integrate across the board—because they share a common goal… But we integrated researchers, we integrated flight controllers, we integrated managers, and it was a necessary thing to do before we actually started the International Space Station.”* – Dunbar (1998, 16 June), astronaut.

In fact, as the following quote from David C. McGill illustrates, since the beginning of NASA’s space program, spaceflight missions have involved large and complex systems integrating different areas of expertise:

*“Building large systems is very much a team sport. It takes a lot of people to do it that range all the way from the architects at the top to the software developers and procurement organizations. There’s a large number of people involved, and there’s decisions being made all up and down this hierarchy.”* – McGill (2015, 22 May), MCC Lead System Architect.

McGill goes on to further discuss the challenges of communicating across a large network of individuals collaborating on a project, while communicating ambiguous demands to all involved. The challenges of arriving at effective and flexible solutions, discussed throughout the interview, characterize much of spaceflight.

**FIGURE 2:**
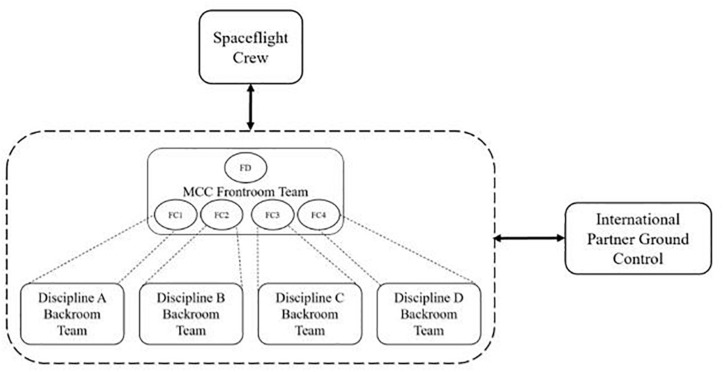
Simplified depiction of NASA’s MCC MTS structure. MCC frontroom team is comprised of the flight director (FD) and flight controllers (FC). Dashed lines indicate supporting relationships between FC and disciplinary backroom teams. Relationships between the MCC MTS and outside teams are depicted as solid double headed arrows.

Originally influenced by military organizations, NASA organized its early structures using a hierarchical structure of specialized teams reporting to a central authority. Within MCC, this structure is comprised primarily of *frontroom* and *backroom* teams. Specifically, the MCC is organized into several disciplines, each assuming responsibility for a hardware system or a specific aspect of the vehicle and mission. Each discipline is represented on the frontroom team by a *flight controller*, who is a discipline specialist. The appointed leader of the frontroom team, overseeing and coordinating all flight systems, is called the *flight director*. During a mission, the flight controllers monitor their assigned system using telemetry data from the vehicle and direct radio communication with the crew. Each flight system’s frontroom flight controller is supported by additional personnel in that system’s backroom team. Given this interdependent arrangement of teams, NASA’s MCC operates as a smaller MTS embedded in the broader SFMTS involved in a mission. Figure 2 provides a simplified depiction of the MTS structure *within* the MCC.

**FIGURE 3:**
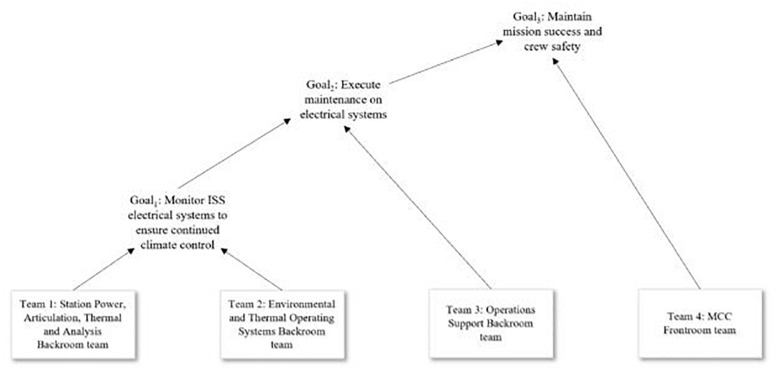
Example goal hierarchy within MCC during an ISS expedition with a need for integration of efforts between frontroom team (Team 4) and backroom teams (Teams 1–3).

The SFMTS structures and relationships in these systems are governed by the nature of the goals pursued by constituent members and teams. That is, constituent members and teams complete different proximal (e.g., individual-level, team-level) goals, which contribute to the overall, superordinate goal of the system (Mathieu et al., 2001). The accomplishment of the superordinate goal (mission success and crew safety, in the case of MCC) requires interdependent interactions among the component teams. In pursuit of this superordinate goal, the component teams within the system will exhibit some form of *functional process interdependence*, meaning that the component teams must work interdependently while accomplishing goals. The exact form and nature of this interdependence will vary according to the needs of the system, and may change over the course of a given mission. An example of a goal hierarchy within MCC is depicted in Figure 3, using the console positions presently in use with the ISS.

National Aeronautics and Space Administration’s front room team serves as a hub for the integration of information from wide ranging disciplines within the organization. Internally, backroom personnel typically communicate with their flight controller on the frontroom team; information passed between backroom teams is most often routed through their respective flight controllers, who confer directly. These interactions are represented in Figure 2 by the dashed lines within the MCC. The backroom teams are located in separate rooms from the frontroom team of flight controllers. Communication between frontroom flight controllers and backroom flight controllers occurs through audio and computer-based methods including email and internal web pages.

This SFMTS structure remains the basis for the organization of MCC, although the composition of the MTS and the distribution of tasks within it have shifted in response to the needs of the missions at the time. Under the present SFMTS organization, crew and frontroom teams must interact efficiently to share information on current and upcoming states of the crew and their taskwork. The discretionary monitoring of this information sharing is largely in the hands of the flight director to determine, a decision role which has notably shaped communication in the midst of past crisis events. Effective communication between the backroom and frontroom team is critical, to ensure that information is effectively transmitted from the backroom teams through to the crew as needed and in a timely manner.

In addition to the frontroom and backroom team interactions, MCC teams interact with the spaceflight crew, with other teams within the broader organization (e.g., management teams), and in more recent years (see findings related to Research Question 2), with teams from international partner (IP) organizations. Frontroom flight controllers are usually the only members of MCC who communicate directly with IP flight controllers or with the crew. Information originating within the backroom teams that must be transmitted to the crew is therefore first relayed through the frontroom team. These patterns of interactions (indicated by solid double-headed arrows in Figure 2) shape and restrict the coordination actions taking place within the SFMTS.

### Research Questions 2 and 3: What Are the Major Goals, Events, and Challenges and How Have NASA’s SFMTSs Adapted?

In order to address our second research question (i.e., *What major goals, events, and challenges have NASA’s SFMTSs encountered?*), our coding team began by identifying the key features of each of the events and/or missions described in the focal JSC OHP transcripts. We also searched for commonalities across the events/missions. Through subsequent discussions with NASA SMEs, our coding team determined that the spaceflight missions undertaken over the past 60 years of the space program can be organized into three distinct eras: (1) *Early Exploration*, (2) *Experimentation*, and (3) *Habitation*. These eras are distinguishable by the goals, events, and challenges encountered by SFMTSs during each period. Table 2 identifies the manned spaceflight programs within each era. Table 3 summarizes the major goals, events, and challenges. With regard to our third research question (i.e., *In what ways have NASA’s SFMTSs adapted over time in response to evolving goals, events, and challenges?*), we determined that during each of the three eras, the SFMTSs exhibited adaptations which corresponded to the major challenges the systems encountered (summarized in Table 4). These adaptations were centered primarily around shifts and/or enhancements in: (1) *technical expertise;* (2) *internal relationships*; and/or (3) *external partnerships*. The following sections provide narrative descriptions of the major goals, events, challenges and adaptations within the three eras.

**TABLE 2:** Spaceflight eras and corresponding NASA programs.

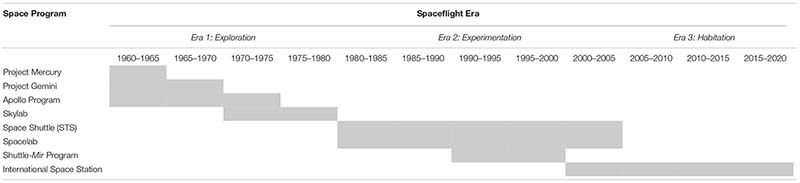

*Shaded areas denote the time period in which a program was active.*

**TABLE 3:** Major goals, critical events, and key challenges within three eras of spaceflight (Research Question 2).

**Era 1: Early Exploration (1960–1980)**	
*Major Goals/Objectives*	• Establish the technical competency needed to overcome the fundamental challenges of spaceflight • Compete effectively with international rivals (“Space Race”)
*Critical Events/Mission Milestones*	• First manned orbital flights (Project Mercury) • Development of intra-lunar manned spacecraft (Project Gemini) • Moon landings (Apollo Program) • Loss of the AS-204 crew (Apollo 1 fire) • Apollo 11 moon landing • Apollo 13 “successful failure” • Launch and maintenance of the Skylab station
*Key Challenges*	• Rapidly overcoming basic challenges of manned spaceflight while *competing* internationally • Overall, progression was anticipated (e.g., Mercury and Gemini programs centered primarily around development of technical capabilities; Apollo missions were the culmination of that development) • However, unforeseen setbacks occurred (e.g., Apollo 1 fire, Apollo 13 explosion)	
**Era 2: Experimentation (1980–2005)**	
*Major Goals/Objectives*	• Capitalize on the technical advancements of the previous era to engage in a program of scientific experimentation in space (international competition no longer a key issue)
*Critical Events/Mission Milestones*	• Space shuttle development and missions (STS) • Hubble telescope maintenance in orbit • Loss of Shuttle Challenger • Loss of Shuttle Columbia • Shuttle-Mir Program/Phase I
*Key Challenges*	• Highly complex and technically challenging missions • Notable performance decrements occur as the result of rigid, unclear, and inefficient communication structures; these decrements presented an unanticipated area of challenge	
**Era 3: Habitation (2000-present)**	
*Major Goals/Objectives*	• Create and maintain an orbital platform to support continuous human occupation. • Collaborate with an array of international partners to accomplish this shared superordinate goal.	
*Critical Events/Mission Milestones*	• The establishment of the International Space Station (ISS) program • The component launches and orbital assembly of the International Space Station • Multiple missions executed in support and supply of the station • Retiring of the space shuttle program • Increased integration of private partnerships for the supply and maintenance of the station • Expedition missions of unprecedented duration (approximately a year in the longest cases)
*Key Challenges*	• Much longer duration missions (presents both technical and interpersonal challenges) • Work successfully with international partners with different norms and work processes • Most of the challenges during this period were not unexpected, but were persistent and critical (e.g., relations between international partners must be maintained continually)

**TABLE 4:** Key SFMTS adaptations across three eras of spaceflight.

**Era 1: Early Exploration (1960–1980)**
***Summary of Adaptations***: NASA’s SFMTSs met the technical competency and external competitiveness demands of Era 1 by establishing and emphasizing formal hierarchies and formalized communication, technical training, and planning procedures
***Examples:***
• Established MTS structures based on military organizations.
• Established communication processes leveraging technology (e.g., vacuum tube messages; headsets).
• Established new training procedures – focused particularly on taskwork (e.g., high-fidelity simulation training for both crew and the ground control teams).
• Established contingency planning procedures – by the time of the Apollo missions there was an emphasis on planning for all eventualities and rehearsing/training these scenarios.

**Era 2: Experimentation (1980–2005)**

***Summary of Adaptations:*** NASA’s SFMTSs evolved to meet the added complexity of Era 2 task demands by shifting their internal communication, collaboration, and oversight structures and practices.
***Examples:***
• Communication processes and structures (particularly internally) changed substantially, in response to unexpected failures.
• Center directors were empowered to make more direct contact with NASA management.
• An Independent Technical Authority was established to make impartial judgements of launch readiness.
• The responsibility of all component teams and contractors to raise concerns related to crew safety or launch readiness was reaffirmed, and reporting practices were articulated.
• Training practices now included additional information about communication and coordination processes – ground control teams received updated training on reporting practices based on the recommendations of the Challenger and CAIB reports.
• Initial steps toward greater collaboration with the Russian space agency made during Shuttle-Mir program; the number of personnel trained to speak Russian and coordinate with international partners began to increase toward the end of this era.
• Technical practices (taskwork training, contingency planning) established during the previous era were refined and expanded.

**Era 3: Habitation (2000-present)**

***Summary of Adaptations:*** NASA’s SFMTSs evolved to meet the challenges of multinational collaboration and long-term habituation within Era 3 by enhancing external communication and collaboration structures and practices.
***Examples:***
• Frontroom team elements comprised of international partner flight controllers were integrated directly into the NASA and ROSCOSMOS frontroom teams.
• NASA crew members learn to speak Russian prior to transport to the station to aid in communication with crewmembers.
• Substantial improvements to interagency communication practices/procedures.
• Enhanced teamwork training procedures to facilitate shared understanding, collaboration, etc.

### Era 1: Early Exploration

#### Major Goals

In the first era, *Early Exploration*, missions including Projects Mercury, Gemini, and the Apollo Program were focused on early forays into space exploration, and required rapid improvements in technical expertise. Further, an intense environment of international competition with rival states (often referred to as the “Space Race”) during the Cold War factored prominently in the motivations and goals of this era. Beginning with early achievements in flight beyond the Earth’s atmosphere (e.g., Shepard’s, 1961 Mercury flight) and continuing through the lunar landings of the Apollo missions and the early forays into extended space habitation through the Skylab station, the superordinate goals pursued by NASA’s SFMTSs centered on developing and applying a significant corpus of technical expertise in a very short period of time in an environment characterized by uncertainty and competition. William Anders captured this focus on exploration and the development of technical expertise in his oral history, and conveyed the extremely uncertain nature of spaceflight at this time:

*“I didn’t think it was risk free but I thought that the [national] reasons for doing it were important, [as well as] the patriotic and… exploration… [This] all made me decide that… there was [probably] one chance in three that [we] wouldn’t make it back, that there was probably two chances in three that we wouldn’t go there either because we didn’t make it back or [we had to abort] and one chance in three we’d have a successful mission, [that this was a risk worth taking].”* – Anders (1997, 8 October), Apollo 8 Lunar Module Pilot.

#### Critical Events, Challenges, and Adaptations

Era 1 was marked by a number of prominent events, including the first manned orbital flights (the focus of Project Mercury), the development of the first effective intra-lunar manned spacecraft (the chief goal of Project Gemini), and the six successful moon landings (the focus of the Apollo Program). These events represent a planned progression from early orbital flight to manned lunar landings. The challenges and successes described during this era related to discovering a need to build and, subsequently, master an expanding body of technical expertise in the realm of spaceflight. In addition, this era was marked by unexpected events that prompted significant adjustments within the system, notably the Apollo 1 fire and the “successful failure” during Apollo 13.

The severe physical and technical challenges inherent to early exploration strained NASA’s capabilities throughout the first era. Tasked with operating in an unfamiliar environment, NASA personnel needed to collaborate intensively to arrive at novel solutions, often in response to problems that were unforeseen at the outset of the mission. In many cases, these challenges were addressed successfully. Nonetheless, this era was also marked by significant failures and tragedies aboard American space vehicles. In many cases, the failures engendered significant changes, improvements, and/or adaptations during subsequent missions.

Prominent among the tragedies driving change within this period is the on-board fire and subsequent total loss of the Apollo 1 (AS-204) crew. During a preflight rehearsal on January 27, 1967, a fire broke out in the cabin of the Apollo 1 Command Module, resulting in the death of all three crew members (astronauts Grissom, White, and Chaffee). Failures in basic protocol as the disaster unfolded revealed critical weaknesses in the planning of missions and tests.

In response to the AS-204 fire, NASA conducted a formal inquiry into the incident, under the *Apollo 204 Review Board*. The report of the board concluded that among other major causes of the accident, emergency preparedness during the test had been inadequate because of the unfueled condition of the rocket and perceived low risk of the test. the disaster instigated a change in the behavioral procedures of NASA. On the day following the disaster, flight control operations branch chief Gene Kranz issued what is now known as the “Kranz Dictum,” which would come to exemplify the future identity of MCC. Kranz is quoted in part as having delivered the following words in response to the disaster:

*“From this day forward, Flight Control will be known by two words: ‘Tough’ and ‘Competent.’ Tough means we are forever accountable for what we do or what we fail to do… Competent means we will never take anything for granted. We will never be found short in our knowledge and in our skills.”* – Gene Kranz, Flight Director, 28 January, 1967.

Kranz’s specified focus on Flight Control as being “tough and competent” directed a continuing tradition of accountability, teamwork, and technical mastery that would continue to mark MCC throughout NASA’s subsequent history. The first adaptation made by MCC, in response to this episode, was a clear delineation of component team responsibilities and accountability. As Kranz’s quote emphasizes, teams and individuals within the system were to be directly accountable for the systems under their control. Combined with the functional specialization of frontroom and backroom teams established early in MCC’s history, this responsibility directed individual component teams to work collectively to support the overall success of the mission, while directing their own internal efforts toward the success of their respective systems. The central issue of accountability and control over launch progress would continue to be a point of struggle for MCC during future missions, as the later loss of the *Challenger* and *Columbia* Shuttles would show. Nonetheless, the incorporation of this lesson following the AS-204 fire represents a critical turning point in the history of MCC.

In contrast to the Apollo 1 fire, the Apollo 13 emergency represented a successful response to an unforeseen technical challenge that required MCC teams to collaborate extensively with a spaceflight crew to arrive at a novel solution. Dubbed a “successful failure” by NASA, the retrieval of the Apollo 13 crew following this severe failure evidences MCC’s growing technical competency. On April 14, 1970, an oxygen tank aboard the Apollo 13 spacecraft exploded. The chaotic atmosphere following the explosion is captured by flight director Glynn Lunney:

*“I [returned to the frontroom] and plugged in at the flight director console to hear a confusing array of multiple indications of problems… The fact of a really serious condition began to dawn on the team as the crew reported the spacecraft venting particles as seen out the window… EECOM was concluding that this was not an instrumentation problem and two fuel cells were indeed lost.”* – Lunney (2010), Flight Director.

The subsequent days required substantial innovation on the part of both the crew and ground teams, perhaps shown most memorably in the construction of the “mailbox” device to aid in removing carbon dioxide from the Lunar Module (LM). In spite of significant technical challenges in even voice communication with the crew, MCC frontroom teams were able to collaborate with both the spaceflight crew and backroom support teams to develop and implement this solution.

The contrast between the AS-204 disaster and the “successful failure” of Apollo 13 highlights a second adaptation instituted within MCC and NASA more broadly. In the years prior to Apollo 13, NASA and MCC had engaged in significant contingency planning and simulation training. The crew’s use of the LM as a “lifeboat” represents an observable outcome of increased planning and preparation, as it had been rehearsed during a training simulation despite the perceived unlikelihood of the plan’s implementation. This contingency planning and simulation reduced the demands on interteam coordination within the system, allowing teams to respond to unfolding events quickly and effectively, without the need to rely on time-consuming direction from central leadership. This freed up communication channels between teams to focus on the transmission of new information, a critical factor in the system’s success.

Representing a third adaptation during this era, rapid communication between component teams and reliance on the largely independent operations of MCC backroom teams allowed MCC personnel to rapidly develop solutions to complex unfolding problems over the course of Apollo 13’s return to Earth. Glynn Lunney captures this developing ability to rapidly respond to new information:

*“The MCC pipeline was regularly delivering a number of new and non-standard checklists for required activities. There were some very effective leaders of specific areas and probably hundreds of operations and engineering personnel evaluating all options and astronaut crews testing each procedure in the simulators.”* – Lunney (2010), Flight Director.

As NASA advanced through Era 1, SFMTSs continued to capitalize on accrued technical and behavioral expertise. This leveraging of technical competency resulted in the first successful lunar landing during the Apollo 11 mission in 1969, as well as five subsequent successful lunar landings. In many ways, the base structure of MCC established during this era has not changed until the present day. The missions MCC has been tasked with supporting over the course of NASA’s history have continued to place similar demands on knowledge integration and coordination of efforts among diverse personnel that prompted the organization of MCC as an MTS initially.

#### Summary of Era 1 Adaptations

As Table 4 summarizes, during Era 1, NASA adapted primarily to meet the technical competency and external competitiveness demands of the period by establishing and emphasizing *formal* hierarchies, communication, training, and planning procedures. Early in this era, NASA adopted rigid, hierarchical organizational structures—and the initial use of the MTS structure—to remain decisive and ensure new information would be rapidly actionable in this uncertain and highly competitive environment. The basic organization of a frontroom team tasked with integrating information among functionally diverse backroom support teams was established early in this era, in response to the technical demands of spaceflight itself. Further, including the role of a flight director as a formalized leadership role within this MTS was recognized as critical to accomplishing the system’s goal of integrating knowledge and coordinating efforts among the various component teams and teams outside MCC.

Additionally, NASA implemented rapid communication practices facilitated by technology (during this era aided by radio headsets and vacuum message tubes), and the extensive documentation of process which is still observable within MCC finds its origins during this first era. Exemplified by the crew’s rapid response to the explosion aboard the Apollo 13 spacecraft described above, MCC personnel acknowledged a need for extensive rehearsal of even unlikely scenarios, given the uncertain nature of spaceflight. Thus, MCC developed extensive training programs which emphasized *technical* competencies and contingency planning to prepare for the uncertain demands of a complex and evolving mission environment.

### Era 2: Experimentation Overview

#### Major Goals

During the second era, *Experimentation*, which included endeavors such as the Space Shuttle missions and the Shuttle-*Mir* Program (i.e., a collaboration between NASA and the Russian space agency ROSCOSMOS), the tasks conducted aboard the spacecrafts became more complex. During this period NASA’s SFMTSs’ efforts centered around capitalizing on the technical advancements of the previous era and conducting research in the unique environment of space. Moreover, following the successes of the Apollo Program (and the end of the “space race”), international competition declined as a central focus of the space program. As noted by Joseph Allen in his oral history interview, the transition toward a focus on experimentation in space began prior to the start of the Space Shuttle missions (i.e., during the later years of Era 1), but was slow to be adopted:

*“[Apollo] 14 was Alan Shepard, who wasn’t all that keen on a lot of science. But [for Apollo 15, science] really stuck. We had crew members [who] liked the science, and we had all kinds of new [science] equipment, and it wound up being the first lunar [mission with geological] traverses that involved some serious distances across all kinds of geology in the rover.”* – Allen (2003, 28 January), Apollo 15 Support Crew Member.

#### Critical Events, Challenges, and Adaptations

The launch and maintenance of the *Skylab* station, which was designed to serve as a solar observatory and platform to support scientific experiments, marked a transitional point in NASA’s mission focus and the event which distinguishes Era 1 from Era 2. This transition represents the beginning of a fusion of both the exploratory focus of the first era and the emphasis on experimentation in space, which would come to dominate the second.

Unfortunately, although it was representative of burgeoning confidence in the ability to execute spaceflight successfully, the station was also plagued by technical difficulties beginning with its initial deployment. During launch, a micrometeoroid shield became dislodged, damaging the solar panels intended to supply power to the station. Archival documents revealed that interview subjects largely focused on the technical challenges of the station’s construction, deployment, and maintenance. This is notable in an oral history interview conducted with Arnold Aldrich:

*“The Skylab 1 first flight had the micrometeoroid protection on the outside of the workshop come off during launch, and it took one solar array with it and pinned down the second one, so that the spacecraft got into orbit without thermal protection and with somewhat limited power… So this temperature was a big concern. Both Marshall and Johnson immediately moved out to figure out how we could quickly ameliorate the overheating in the workshop.”* – Aldrich (2000, 24 June), Deputy Manager (Skylab Program).

In spite of these difficulties, maintenance Skylab showcased the increased technical achievement of NASA, with the deployment of a sunshield to prevent overheating and two additional Extravehicular Activity (EVA) repairs being the focus of the first of three manned missions to the station (SL-2).

Although the loss of the station to orbital decay, in some ways, represented the still-present technical challenges faced by NASA, it was also the result of the growing prioritization of the development of the *Space Shuttle Program*, the centerpiece of the second era. The space shuttle program epitomizes the second era. Over the lifetime of the program, the shuttle was used both as an Earth-to-orbit transportation vehicle as well as an orbital experimental platform. Similar to the missions comprising the first era, shuttle missions were short in duration, lasting for days to approximately 2 weeks. To facilitate the experimental mission of the shuttle, a laboratory module called “Spacelab” was sometimes incorporated into the shuttle.

NASA’s increasing focus on experimentation was facilitated in large part by the technical competencies accrued during the previous era. In a revealing passage from a NASA mission archive on STS-61, maintenance on the Hubble Space Telescope is described as being completed ahead of schedule, with a few unexpected events being handled smoothly. This characteristically competent mission completion occurs within the context of “*one of the most challenging and complex manned missions ever attempted*” (Ryba, 2010). Interestingly, following the establishment of the shuttle program, NASA’s objectives of experimentation often differed from those of IPs, as ROSCOSMOS objectives aboard the station focused more on simply maintaining a manned presence in space (Foale, 1998).

However, this era was also characterized by major disasters. One of the greatest tragedies to occur during this era of spaceflight was the loss of the shuttle *Challenger* and its entire astronaut crew (STS-51L). A series of aborted launches due to a range of weather concerns lead to mounting impatience, and an eventual go-ahead for the launch despite concerns over low temperatures. This push to move forward with the launch was exacerbated by plans to widely televise the launch. The conflict between caution and the mounting pressure to launch within MCC is captured by Steve Nesbitt, a NASA public affairs officer working at MCC at the time:

*“There had been a couple of scrubs in the days before. That was not unusual. Some of the most conservative people you will ever find are in Mission Control. If something wasn’t right, they were quite willing to delay and come back another day. But that mission just went on and on.”* – Nesbitt (2016, 28 January), NASA MCC Public Affairs Officer.

Following the loss of the shuttle *Challenger*, President Reagan established a commission to conduct an investigation into the disaster and potential ways in which the disaster might have been averted. The commission concluded that “flaws in (NASA’s) decision making process” were a contributing cause of the accident (Presidential Commission on the Space Shuttle Challenger Accident, 1986). The report found that failures in communication resulting from incomplete and misleading information, in conjunction with a NASA management structure which permitted known safety issues to bypass shuttle managers, led to known risks remaining unaddressed in readiness reviews. In the recommendations provided by the commission, improvements to management and communications factor prominently, with an emphasis on managerial integration and improved communication across the organization (recommendations II and V; Presidential Commission on the Space Shuttle Challenger Accident, 1986, p. 199–200).

In response to the commission’s recommendations, the hierarchy of organization within the Office of Space Flight was restructured to allow the MCC far more direct access to NASA administration. Regular, formalized communication between the directors of JSC and other organizational components were instituted. Perhaps most notably, the accountability of center directors for the “technical excellence and performance of the project elements assigned to their centers” was reaffirmed (Presidential Commission on the Space Shuttle Challenger Accident, 1987, p. 31). These adjustments in the interteam collaboration processes of the MCC represent the first integration of lessons learned based on the challenges of this era.

Despite the implementation of these recommendations, the subsequent loss of the shuttle *Columbia* would illustrate the need for further adaptations in NASA’s internal collaboration. On February 1, 2003, the Shuttle *Columbia* disintegrated while reentering the atmosphere, resulting again in a complete crew loss (STS-107). The failure resulted from damage from foam impacting the wing of the spacecraft during launch. In a subsequent investigation, the Columbia Accident Investigation Board (CAIB) concluded that NASA engineers had raised concerns following the launch that the foam shedding damage to Columbia may have been more significant than in previous launches. NASA managers did not initiate investigations into this possibility. Notably, the report concluded that flaws within the organizational structure of NASA were significant contributors to the disaster, and the loss would likely have occurred irrespective of which individuals were in the managerial roles.

In a second adjustment, following the recommendations made by the CAIB, NASA and MCC implemented several changes to the structure and behavior of MCC (Columbia Accident Investigation Board [CAIB], 2003). Among these changes was the establishment of an independent Technical Engineering Authority, “responsible for technical requirements and all waivers to them” (Columbia Accident Investigation Board [CAIB], 2003, p. 193). In keeping with the recommendations of the CAIB, the technical authority became the sole authority for all technical standards, and independently verified launch readiness with the ability to reject any scheduled launch should an undue risk be found. Critically, the ITA would be funded directly from NASA headquarters, removing it from any, “connection to or responsibility for schedule or program cost” (Columbia Accident Investigation Board [CAIB], 2003, p. 193). The ability of any component team to raise objections about the readiness of any system for launch was also reaffirmed. These changes increased the safety of future shuttle crews by allowing evaluation of launch readiness not subject to constraints or pressures from other elements within the organization.

Despite these two public failures, the program of experimentation in space continued largely successfully throughout the second era. One of the lasting legacies of the shuttle program is the ability to launch large payloads into orbit, which would be critical during the following era. Moreover, beginning in 1995 and continuing through 1998, NASA collaborated with ROSCOSMOS to host American astronauts aboard the Russian *Mir* space station (the Shuttle-*Mir* Program). Accordingly, astronauts conducted research aboard the orbital platform while the space shuttle continued to be used for resupply and crew transport. During this program, sometimes called *Phase I*, NASA MCC personnel learned to form conducive working relationships with Russian ground control teams, requiring them to overcome challenges arising from language and cultural barriers (Reeves, 2009; Hill, 2015).

However, international collaboration was undoubtedly affected by external socio-political forces. For example, the fall of the USSR in 1991 led to improved relations between the Russian Federation and the United States, and a corresponding increase in the potential for international collaboration. The 1992 agreement between Presidents Bush and Yeltsin solidified plans for cooperation in space exploration, leading to the Shuttle-*Mir* and subsequent programs, although relations between organizations from the two countries would remain challenging.

A clear demonstration of these challenges can be found in astronaut Michael Foale’s time aboard the *Mir* station. During that period an unmanned *Progress* spacecraft collided with the station, causing substantial damage and a fire aboard the station. Despite initial trepidations among the Russian ground teams, Foale was allowed to take part in EVAs to repair the station following the development of a medical issue by cosmonaut Tsibliev. Accomplishing this goal required MCC personnel to coordinate rapidly with Russian ground control (TSuP) to secure permission for Foale to conduct the EVAs, as well as effective coordination among both ground control groups and the international members of the crew to quickly familiarize Foale with the Russian-made EVA equipment (Foale, 1998).

#### Summary of Era 2 Adaptations

During Era 2, NASA’s SFMTS adapted to meet the added complexity of task demands by improving internal communication, collaboration, and oversight structures and practices. NASA personnel were empowered to raise concerns in connection with launch readiness directly; the responsibility of all NASA personnel to raise such concerns as they became aware of them was reaffirmed. Training procedures introduced during this era targeted effective internal communication practices directly. Finally, an Independent Technical Authority was established to make impartial judgments about launch readiness, outside the NASA managerial hierarchy.

Where failures occurred, they prompted adaptations to coordination within MCC and the SFMTS. Where challenges were successfully addressed, the outcomes exemplify critical competencies built during the first era of spaceflight: extensive contingency planning, leveraging of large amounts of training to arrive at innovative solutions, and rapid communication among functionally diverse teams. In spite of these successes, structural weaknesses within the MCC resulted in failures during this era, requiring further changes to be made in order to prevent future breakdowns in process.

As was the case during Era 1, SFMTS adaptations in Era 2 were often prompted by unexpected external events—in this case, often socio-political ones. In particular, the challenges in coordination between teams from NASA and ROSCOSMOS demonstrated an increasing need for familiarity both with IP equipment and practices, a need which led to the introduction of more extensive SFMTS training within the subsequent era of habitation. As a result, during the Shuttle-*Mir* program, NASA’s MCC evolved in their ability to coordinate effectively with IP organizations. In fact, the MCC MTS expanded to include remote personnel embedded with Russian ground control teams. These international consulting teams represented an early advancement in formalizing the relationship between NASA MCC and Russian ground control personnel, a challenge which would continue to be addressed during the subsequent era of habitation. Subsequently, the success of the Shuttle-*Mir* program laid the groundwork for the International Space Station program—and the increasingly intense international collaborations that would be required by that program. This transition is highlighted in Dr. Michael Barratt’s oral history interview:

*“Those of us that were heavily involved in the Shuttle-Mir Program realized two things. How wonderful it would be, because we found that we could work with our Russian counterparts quite well, and how difficult it would be, because they do things very differently than we do… Without the Shuttle-Mir Program I can’t imagine starting from scratch and going into such a large program as the International Space Station*” – Barratt (1998, 14 April), Human Research Program Manager.

### Era 3: Habitation

#### Major Goals

In the third era, *Habitation*, which consisted primarily of the construction of and expeditions aboard the International Space Station (ISS), mission objectives centered on establishing a continuous human presence in space in collaboration with IP organizations. The major goal of Era 3 was the construction and maintenance of an orbital platform to support continuous human occupation. The primary operational difference between the activities of Era 3 and earlier periods is the extended mission timeframe of ISS expeditions. The ISS has been continuously inhabited since late 2000, with the longest individual crew member stays lasting approximately 1 year.

#### Critical Events, Challenges, and Adaptations

The challenges facing SFMTSs during Era 3 centered on overcoming difficulties related to international collaboration and the physical challenges of long-duration spaceflight. In Era 3, NASA has needed to collaborate intensively with an array of IPs in pursuit of shared goals. Moreover, whereas previous eras were characterized by missions lasting several days, this era is marked notably longer spans of habitation aboard the ISS (e.g., 6 months).

To support the station, the MCC has engaged in continuous operations for 18 years. This shift from short-duration, high-intensity missions to a long-term mission timeline requires MCC to operate in fundamentally different ways than they did during prior missions and eras of spaceflight. New skills relevant to the monitoring and maintenance of the crew and station have become more salient to the present task, shifting the needs of the system in important ways. Additionally, extended habitation in space places immense strain on astronauts’ bodies, including loss of visual acuity, muscle loss, and loss of bone density. In turn, these physical challenges can exacerbate the already intense psychological strain on astronauts. Combined with the challenges of existing for a prolonged period of time in a confined space alongside a diverse, international crew, the confluence of these psychological strains can be intense. The challenges of intensive collaboration with IP organizations are discussed by Dr. Michael Barratt during his 2015 interview for the International Space Station oral history project:

*“I think if anybody had asked us what a good model for making a Space Station would be, the answer would not have been to choose a major partner who speaks another language, who uses metric system rather than English system, who has a totally different engineering philosophy, safety culture, methods of operation, methods of manning. All of that was different.”* – Barratt (2015, 30 July), Human Research Program Manager.

The types of challenges described by Michael Barratt in the above quote required NASA and their IPs to leverage the lessons of the previous two eras of spaceflight. As in the era of experimentation, NASA’s SFMTSs in the third era have continued to draw on the technical competencies built during prior eras. Michael Barratt further discusses technical competency in the context of the ISS, with respect to the ISS’s usage as a platform for scientific experimentation:

*“I think one of the main things is that just looking at the Station as a laboratory, it has grown in capability, and it enables science that we could never do before, because it is power-rich, and it has an incredible bandwidth to it… the laboratory that [the ISS has] evolved into is just incredibly capable.”* – Barratt (2015, 30 July).

These competencies were combined with the capabilities for launching large orbital payloads developed during the era of Experimentation. Leveraging this knowledge and the lessons of the Shuttle-*Mir* program, NASA collaborated closely with a wide range of IPs to complete the ambitious ISS platform in 2011. As summarized by Michael Suffredini, the legacy of the ISS is to consciously build and demonstrate capabilities to sustain human habitation in space for extended periods of time.

*“The legacy of ISS will be that we created an environment that allowed us to permanently have humans in low-Earth orbit. That, by its very nature, will mean that the ISS helped us do exploration, because we have the capability permanently in low-Earth orbit to do the things we need to do to safely travel beyond low-Earth orbit.”* – Suffredini (2015, 29 September), ISS Program Manager.

Accordingly, NASA SFMTSs have had to develop substantial procedures for coordination among IP ground control teams in order to meet the challenges of international collaboration in spaceflight, as well as building a number of technical competencies to facilitate this relationship. Representing a first adjustment during this era, over the course of the Shuttle-*Mir* program and subsequent phases of the ISS project a large number of NASA engineers learned Russian (Barratt, 1998), and channels of communication were established which grew more developed as communication technologies advanced and communication between the organizations normalized (Reeves, 2009; Hill, 2015). Among these adaptations were the inclusion of a Russian console in MCC, as well as a translator loop allowing MCC flight controllers to listen in on the communications between the Russian ground control teams and their crew members aboard the station. Dr. Barratt discusses this finding of common ground in his oral history interview.

*“Once you get past the language barrier, people understood that the laws of physics are the same, the laws of orbital mechanics are the same, zero gravity is the same, and it was pretty easy to find common ground amongst the crewmembers and the supporting engineers. Really language was the only thing in the way there. A lot of United States engineers learned Russian, a lot of Russians learned English, which was quite wonderful. Once we got through that, we found that we could work together pretty well.”* – Barratt (2015, 30 July), Human Research Program Manager.

Lastly, the challenges in terms of interteam relations between teams in MCC, other NASA teams, and IP teams have resulted in the integration of interpersonal and team skills training into the training regimen of astronauts and flight controllers. Notably, the present iterations of these training practices focus primarily on enhancing teamwork within *individual* teams, rather than teamwork processes spanning across multiple teams.

#### Summary of Era 3 Adaptations

Adaptations made during this era centered around meeting the challenges of multinational collaboration and long-term habitation by developing greatly improved *external* collaboration practices. Altered practices and competencies aided in more rapid and effective communication across organizational and national boundaries, as did dedicated training in teamwork practices. Interventions aimed at teamwork helped ensure that the multinational crew aboard the station was able to function effectively, and interpersonal conflict resulting from the challenging physical and relational environment was minimized.

## Discussion

Drawing from archival sources, this case study identified many of the collective memories (e.g., mission successes, failures), lessons learned, and adaptations or practices implemented within NASA’s SFMTSs in the three prior eras of early exploration, experimentation, and habitation. NASA and their IPs are now on the brink of an anticipated *fourth* era of spaceflight, characterized by LDEMs. The “team risk” will play a much larger role than in previous missions, as team and interteam coordination must be sustained for *multiple years* as SFMTSs tackle unexpected and even dangerous challenges (Salas et al., 2015). We expect that whether these systems will be able to address the challenges of future missions will be impacted by the rich history of the organizational environment, the lessons learned in previous missions, and the organizational practices related to teamwork that have been implemented within NASA.

### Synthesizing the Adaptations of Previous Eras to Facilitate Adaptive Performance in the Next Era of Spaceflight

As summarized in Table 4, our analysis of archival documents revealed three broad categories of adaptations used to meet the evolving task demands of the previous eras of spaceflight: (1) *enhancing technical expertise*, (2) *enhancing or shifting internal collaborative relationships*; and (3) *enhancing external or cross-organizational partnerships*. Interestingly, we find that NASA’s SFMTSs emphasized these different categories of adaptations in different ways within each era. During Era 1, the external competition and the massive demands for improved technical competence meant that the primary focus was on enhancing *technical* expertise. In Era 2, NASA complex mission demands continued to require new technical developments, however, unexpected disasters (e.g., the losses of Challenger and Columbia) revealed that adaptations were urgently needed with regard to *internal collaboration* patterns. Lastly, in Era 3, the installation of the ISS necessitated a focus on *external partnerships* with international agencies.

**FIGURE 4:**
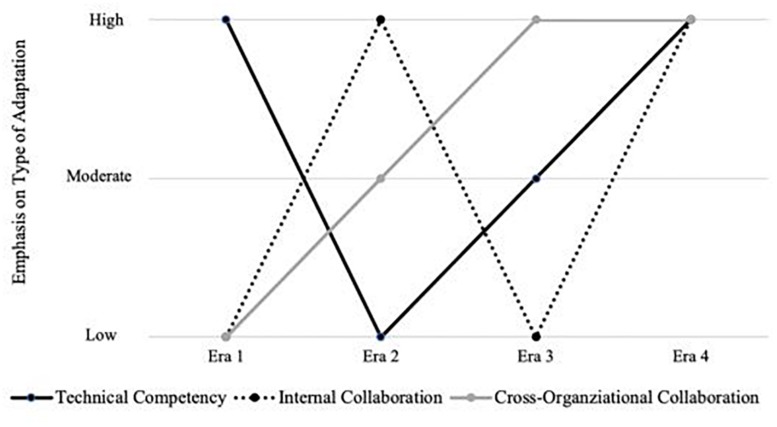
Amount of emphasis on different types of adaptations with each spaceflight era. Emphasis varied across eras with regard to (1) enhancing technical competencies (solid black line); enhancing internal collaboration (dashed black line); and (3) enhancing cross-organizational partnerships (gray line).

Figure 4 summarizes the emphasis on different categories of adaptive behaviors across the previous three eras. As we enter into the fourth era of spaceflight exploration, NASA’s SFMTSs must not lose the gains made in previous eras. The challenges of LDEMs reflect those seen within early exploration, experimentation, and habitation. However, LDEMs also present new challenges that will call for new adaptations. Indeed, as shown in Figure 4, NASA’s SFMTSs will need to significantly enhance their technical capabilities, internal collaborative relationships, and external partnerships in order to achieve the goals of LDEMs.

In the following, we discuss the anticipated challenges of the upcoming era of human spaceflight, and the adaptations that will be required. Given the complex challenges involved in LDEMs, NASA’s SFMTS will need to adapt substantially across all three domains (i.e., technical expertise, internal coordination, and external coordination). This need to reconsider existing practices in the light of new challenges is nothing new to NASA, as our review demonstrates. For example, in an oral history interview conducted in May of 2015, MCC lead system architect David McGill states:

*“Well, how will your design react if suddenly we have a mission that is going to involve three countries to go fly it? How are you going to tolerate that? How is your system going to respond to all of a sudden wide area networking is twice as fast and half as much money as it is today? Can you take advantage of that?”* – McGill (2015, 22 May), MCC Lead System Architect.

First, echoing Era 1, LDEMs will bring demands for adaptation in *technical* expertise. For example, the distances to be traveled in LDEMs represent a significant technical challenge. A variety of technical approaches to manned Mars missions and other LDEMs have been discussed (e.g., the Lunar Gateway platform; National Aeronautics and Space Administration [NASA], 2014); but all will require substantial technical advancements. Further, the distances involved in LDEMs will require extremely long periods of travel beyond which will place new strains on astronauts. Negative physical effects may become continuously more severe over the greater mission timeframes of LDEMs. The extended time the crew will be isolated from the rest of the system leads to particularly intense concerns around training retention, as technical training is known to degrade over time and the highly autonomous crew will be less able to rely on support from ground-based teams (Landon et al., 2018).

The challenges of LDEMs will also require adaptation with respect to *internal* collaboration practices. As an unavoidable consequence of the massive distances traveled during a LDEM, there will be significant communication delays between the spaceflight crew and earthbound teams. At the greatest distance, communications to or from the crew of a Mars mission could take up to 24 min to arrive at their destination. Such communication delays represent a stark contrast with the effectively instantaneous communications between MCC and the crew of the ISS. In the third previous eras of spaceflight, crews relied heavily on rapid communication with Earthbound teams to arrive at solutions. However, in LDEMs, the crew will need to operate far more independently, as reliance on continuous feedback from MCC will not be feasible. Such *decentralized* authority structures may be necessary for LDEM success but may also present challenges for multiteam coordination and performance (Lanaj et al., 2013).

Finally, the upcoming era of spaceflight will require continued adaptation in the domain of *external* coordination and collaboration. LDEMs will reach further than any prior manned spaceflight mission and will require massive inter-agency coordination across national and organizational borders. The SFMTSs involved in LDEMs will be comprised of members from different cultures, backgrounds, nations, and areas of expertise. Such high levels of individual and team differentiation are likely to pose challenges for interteam collaboration (Luciano et al., 2018). Moreover, SFMTSs involved in LDEMs will experience dynamic environments characterized by expected (e.g., increased communication delays) and unexpected challenges. As a LDEM progresses, different areas of technical expertise will become more or less relevant to the task at hand, resulting in shifts in goal priorities and the relative authority of teams over the course of the mission. As these responsibilities may be distributed across IPs teams (as with the current operation of the ISS) these highly dynamic contexts may exacerbate tensions surrounding organizational boundaries and hinder communication and interteam coordination (Luciano et al., 2018).

Moreover, the consequences of longer-duration mission timelines for internal and external collaboration remain in question. Whereas research on team tenure would seem to suggest that performance of the system will increase over time (Bell, 2007), initial evidence from research conducted using NASA analog environments has demonstrated that when crews are restricted to isolated environments for prolonged periods of time, longer team tenure can lead to collaboration and cohesion decrements as interpersonal conflicts becomes more severe (Kozlowski et al., 2016). Indeed, concerns have been expressed around the strain that long-duration spaceflight may place on astronauts and the potential negative effects for interpersonal relations both within the crew and across component teams in SFMTS (Palinkas, 2007; Palinkas and Suedfeld, 2008; Landon et al., 2018).

### Beyond LDEMs: Theoretical and Practical Contributions

This case study is focused on the specific context of NASA’s SFMTSs. However, there are at least four ways in which the findings from this research might inform MTS research and practices within other contexts. First, our review revealed that adaptations with were driven by the focus and challenges of the periods in which they were enacted and clustered into one of three general categories: (1) technical competency, (2) internal coordination, and (3) external or cross-organizational coordination (see Figure 4). Although the adaptations identified in archival documents were generally specific to NASA, the three-category framework may be useful for conceptualizing and advancing MTS adaptations in other contexts. With respect to MTS research, future empirical work may benefit from the greater specificity of these dimensions, and their relationship with situational and task demands. In practice, organizations can target the dimensions of adaptation that have successfully addressed related challenges in the past when preparing for upcoming challenges. In particular, anticipating the needed patterns of adaptation may allow for more successful proactive intervention–thus avoiding the inefficiencies of adapting after needs are revealed by performance decrements. Strategies allowing for more successful proactive adaptation are especially relevant to high-reliability organizations operating in dynamic environments (HROs) like NASA, the military, and disaster response teams. HROs often operate in unforgiving competitive, social, and political environments that are rich in potential for error, and where the scale of consequences associated with error precludes learning through experimentation (Weick et al., 1999).

Second, consistent with prior theoretical work on MTSs (e.g., Zaccaro et al., 2012), our case study revealed compositional and linkage attributes that factor prominently in the functioning of SFMTSs. For example, our review established that component teams in the MCC (i.e., frontroom and backroom teams) are highly differentiated along a variety of dimensions (e.g., areas of expertise, work processes, geographic locations). Although team differentiation is a necessary element of MTS collaboration which allows these systems to divide complex interdisciplinary tasks into disciplinary subgoals, the extreme levels of differentiation often seen in SFMTSs can also incur performance decrements when relationships are not managed effectively (Luciano et al., 2018). In fact, whereas the SFMTSs within Era 1 emphasized formal structures and separations between teams, in order to tackle new demands in Era 2, the SFMTSs began to permit more direct communication channels between people who were otherwise disconnected (e.g., occasional guidance from specialists to crewmembers conducting experiments). These findings suggest an interesting line of inquiry for MTS researchers–MTSs may need to strike the right balance in terms of emphasizing component team separation and integration. However, the optimal balance point may vary based on evolving task demands.

Third, our analysis of the history of SFMTSs suggests MTS research could benefit from considering MTS performance and adaptation on a longer time scale than has been used in previous research. Empirical studies of MTS functioning have focused primarily on performance as a relatively short-term outcome. Although these studies provide valuable contributions to our understanding of MTS functioning, our review of NASA archival documentation revealed that in several cases, short-term failures in performance led to improved performance in the future (e.g., the structural changes made to NASA’s management hierarchy in response to the losses of shuttles Challenger and Columbia).

Our findings also provide insight into how adaptation might manifest in HRO contexts following a performance failure. Unlike many teams in which creative solutions are required (e.g., product development teams), teams and MTSs operating within HROs cannot afford to readily accept short-term failures as a means to facilitating learning and adaptation. Nonetheless, errors and failures in performance are a virtual certainty over the long-term. Our findings indicate that the key to successful adaptation may lie in maximizing the information extracted from the events, and its successful integration into future practices. Illustrating this, NASA conducts unflinching internal examinations following critical events to establish both their immediate and structural causes. Notably such rigorous investigations do not only occur in cases where human life has been lost or placed at great risk; this dedication to intensive examination in the wake of any failure is exemplified by the rigorous investigation following the loss of the unmanned Mars Climate Orbiter (MCO) in 1999 (Mars Climate Orbiter Mishap Investigation Board, 1999). Practices like these may be of benefit to even non-HRO organizations, suggesting a wider application of this approach (Weick et al., 1999).

Lastly, we suggest that our case study approach may be applicable in a range of contexts outside NASA as many teams and MTSs have collective performance experience. This work is in keeping with recommendations to conduct qualitative ethnographic research prior to and following quantitative research within an organization (Ofem et al., 2012). Given the impact of a MTS’s history on its future operations, we expect continued qualitative examinations of this type will serve to better inform LDEMs, and could serve as the foundation for broader explorations of MTS temporal dynamics. These benefits could be further expanded in future research through detailed examination of the day-to-day operations of MTSs, with respect to the enduring effects of these events in the future. Although the need to consider the rich history of an organization is often acknowledged by practitioners, there is also a proliferation of “off-the-shelf” interventions available. This case study may serve as a reminder that anchoring organizational interventions in an understanding of the historical context of the organization may increase their effectiveness.

## Conclusion

In conclusion, scholars have argued that a team’s history can significantly impact its future (Marks et al., 2001; Hollenbeck et al., 2014). Our analysis of the evolution and adaptation of NASA’s history suggests that the same can be said of a SFMTS. We find the lessons learned in previous eras of spaceflight often carry forward into subsequent phases. Our findings revealed that adaptations typically clustered into one of three general categories and were associated with specific types of task demands and critical events. We suggest that LDEM SFMTSs will need to capitalize on the gains of the past while incorporating additional adaptations in order to succeed. Thus, this case study demonstrates the value of examining prior patterns of adaptation in preparation for future challenges.

## Author Contributions

All authors contributed substantially to the identification, classification, and analysis of archival documents and to the development of the conceptual framing of this manuscript. LL and KS contributed as subject matter experts, and aided significantly in the development of a conceptual framework for the classification of archival resources. Finally, all authors contributed significant amounts of time and effort to the revision of the text and the refining of the conceptual and historical content.

## Conflict of Interest Statement

The authors declare that the research was conducted in the absence of any commercial or financial relationships that could be construed as a potential conflict of interest.

## Appendix A

**TABLE A1:** List of sources used in archival analysis.

**Johnson Space Center Oral Histories**		
**Subject**	**Date of interview**	**Retrieved From**
Andrew S.W. Thomas	7/22/1998	https://historycollection.jsc.nasa.gov/JSCHistoryPortal/history/oral_histories/Shuttle-Mir/ThomasASW/ThomasASW_7-22-98.htm
Arnold D. Aldrich	6/24/2000	https://historycollection.jsc.nasa.gov/JSCHistoryPortal/history/oral_histories/AldrichAD/AldrichAD_6-24-00.htm
Bonnie J. Dunbar	1/20/2005	https://historycollection.jsc.nasa.gov/JSCHistoryPortal/history/oral_histories/DunbarBJ/DunbarBJ_1-20-05.htm
Bonnie J. Dunbar	6/16/1998	https://historycollection.jsc.nasa.gov/JSCHistoryPortal/history/oral_histories/Shuttle-Mir/DunbarBJ/DunbarBJ_6-16-98.htm
Bonnie J. Dunbar	3/23/2005	https://historycollection.jsc.nasa.gov/JSCHistoryPortal/history/oral_histories/DunbarBJ/DunbarBJ_3-23-05.htm
Bonnie J. Dunbar	9/14/2005	https://historycollection.jsc.nasa.gov/JSCHistoryPortal/history/oral_histories/DunbarBJ/DunbarBJ_9-14-05.htm
C. Michael Foale	6/16/1998	https://historycollection.jsc.nasa.gov/JSCHistoryPortal/history/oral_histories/Shuttle-Mir/FoaleCM/FoaleCM_6-16-98.htm
C. Michael Foale	7/7/1998	https://historycollection.jsc.nasa.gov/JSCHistoryPortal/history/oral_histories/Shuttle-Mir/FoaleCM/FoaleCM_7-7-98.htm
C. Michael Foale	7/31/1998	https://historycollection.jsc.nasa.gov/JSCHistoryPortal/history/oral_histories/Shuttle-Mir/FoaleCM/FoaleCM_7-31-98.htm
Christopher C. Kraft	6/28/1991	https://www.nasa.gov/sites/default/files/atoms/files/19910628_christopher_kraft_oral_history_interview.pdf
David C. McGill	5/22/2015	https://historycollection.jsc.nasa.gov/JSCHistoryPortal/history/oral_histories/McGillDC/McGillDC_5-22-15.htm
Donald D. Arabian	2/3/2000	https://historycollection.jsc.nasa.gov/JSCHistoryPortal/history/oral_histories/ArabianDD/DDA_2-3-00-amended.pdf
Eugene F. Kranz	1/8/1999	https://historycollection.jsc.nasa.gov/JSCHistoryPortal/history/oral_histories/KranzEF/KranzEF_1-8-99.htm
Gerald P. Carr	10/25/2000	https://historycollection.jsc.nasa.gov/JSCHistoryPortal/history/oral_histories/CarrGP/CarrGP_10-25-00.htm
Glynn S. Lunney	7/16/2010	https://historycollection.jsc.nasa.gov/JSCHistoryPortal/history/oral_histories/LunneyGS/Apollo13.htm
Guion S. Bluford	8/2/2004	https://historycollection.jsc.nasa.gov/JSCHistoryPortal/history/oral_histories/BlufordGS/BlufordGS_8-2-04.htm
Jack R. Lousma	3/15/2010	https://historycollection.jsc.nasa.gov/JSCHistoryPortal/history/oral_histories/LousmaJR/LousmaJR_3-15-10.htm
John W. Aaron	1/26/2000	https://historycollection.jsc.nasa.gov/JSCHistoryPortal/history/oral_histories/AaronJW/AaronJW_1-26-00.htm
Joseph P. Allen	1/28/2003	https://historycollection.jsc.nasa.gov/JSCHistoryPortal/history/oral_histories/AllenJP/AllenJP_1-28-03.htm
Leon T. Silver	5/5/2002	https://historycollection.jsc.nasa.gov/JSCHistoryPortal/history/oral_histories/SilverLT/SilverLT_5-5-2002.pdf
Michael R. Barratt	7/30/2015	https://historycollection.jsc.nasa.gov/JSCHistoryPortal/history/oral_histories/ISS/BarrattMR/BarrattMR_7-30-15.htm
Michael R. Barratt	4/14/1998	https://historycollection.jsc.nasa.gov/JSCHistoryPortal/history/oral_histories/Shuttle-Mir/BarrattMR/BarrattMR_4-14-98.htm
Michael T. Suffredini	9/29/2015	https://historycollection.jsc.nasa.gov/JSCHistoryPortal/history/oral_histories/ISS/SuffrediniMT/SuffrediniMT_9-29-15.htm
Paul F. Dye	6/16/1998	https://historycollection.jsc.nasa.gov/JSCHistoryPortal/history/oral_histories/Shuttle-Mir/DyePF/DyePF_5-27-98.htm
Paul S. Hill	3/24/2015	https://historycollection.jsc.nasa.gov/JSCHistoryPortal/history/oral_histories/HillPS/HillPS_3-24-15.htm
William D. Reeves	6/22/1998	https://www.spaceflight.nasa.gov/history/shuttle-mir/people/oral-histories/reeves.pdf
William D. Reeves	4/17/2009	https://historycollection.jsc.nasa.gov/JSCHistoryPortal/history/oral_histories/ReevesWD/ReevesWD_ 4-17-09.htm

**Official NASA or Government Reports**		
**Report Name**	**Relevant Mission or Program**	**Retrieved From**

Phillip L. Engelauf	6/24/1998	https://historycollection.jsc.nasa.gov/JSCHistoryPortal/history/oral_histories/Shuttle-Mir/EngelaufPL/EngelaufPL_6-24-98.htm
William A. Anders	10/8/1997	https://historycollection.jsc.nasa.gov/JSCHistoryPortal/history/oral_histories/AndersWA/AndersWA_10-8-97.htm
Actions to Implement the Recommendations of The Presidential Commission on the Space Shuttle Challenger Accident	STS-51L	https://history.nasa.gov/rogersrep/actions.pdf
Apollo 13 Mission Report	Apollo 13	https://www.hq.nasa.gov/alsj/a13/A13_MissionReport.pdf
Bilateral agreement between NASA and the Russian Space Agency	ISS	https://www.nasa.gov/mission_pages/station/structure/elements/nasa_rsa.html
Columbia Accident Investigation Board [CAIB] (2003). Columbia accident investigation board report.	STS-107	https://www.nasa.gov/columbia/home/CAIB_Vol1.html (http://s3.amazonaws.com/akamai.netstorage/anon.nasa-global/CAIB/CAIB_lowres_full.pdf)
Implementation of the Recommendations of the Presidential Commission on the Space Shuttle Challenger Accident	STS-51L	https://history.nasa.gov/rogersrep/v6index.htm
Investigation of the Challenger Accident Congressional Report	STS-51L	https://www.gpo.gov/fdsys/pkg/GPO-CRPT-99hrpt1016/pdf/GPO-CRPT-99hrpt1016.pdf
President Nixon’s 1972 Announcement on the Space Shuttle	Space Shuttle Program	https://history.nasa.gov/stsnixon.htm
Report of Review Board on Apollo mission AS-204	AS-204	https://history.nasa.gov/Apollo204/chro.html
Report of the Committee on Aeronautical and Space Sciences, United States Senate with Additional Views – Apollo 204 Accident, January 30, 1968	AS-204	https://history.nasa.gov/as204_senate_956.pdf
Report of the PRESIDENTIAL COMMISSION on the Space Shuttle Challenger Accident 6/6/1986	STS-51L	https://history.nasa.gov/rogersrep/genindex.htm
Seeking a Human Spaceflight Program Worthy of a Great Nation	Human Spaceflight Program	https://www.nasa.gov/pdf/396093main_HSF_Cmte_FinalReport.pdf

**Other Sources**		
**Source Name**	**Relevant Mission or Program**	**Retrieved From**

”Chronology of Defining Events in NASA history”	Various	https://history.nasa.gov/40thann/define.htm
Apollo 13 Lunar Module ’Mail Box’	Apollo 13	https://www.nasa.gov/image-feature/apollo-13-lunar-module-mail-box
Description of ISS modules	ISS	https://www.nasa.gov/mission_pages/station/structure/elements/space-station-assembly
Description of ISS participants and roles	ISS	https://www.nasa.gov/mission_pages/station/cooperation/index.html
Ed White biography; section on Gemini 4 EVA	Gemini 4	https://history.nasa.gov/Apollo204/zorn/white.htm
ESA article on Soyuz MS-09	Soyuz MS-09	http://www.esa.int/Our_Activities/Human_Spaceflight/International_Space_Station/Liftoff_Alexander_Gerst_returns_to_space
History of Shuttle-Mir	Shuttle-Mir program	https://spaceflight.nasa.gov/history/shuttle-mir/Shuttle-Mir_text-only.htm
International Space Station Status Report – ISS98-03	ISS; launch of Zarya module	https://www.nasa.gov/centers/johnson/news/station/1998/iss98-03.html
NASA chronology of Apollo-Soyuz missions	Apollo-Soyuz	https://history.nasa.gov/40thann/define.htm
NASA mission archive on STS-71	STS-71	https://www.nasa.gov/mission_pages/shuttle/shuttlemissions/archives/sts-71.html
NASA-4: Fire and Controversy	Shuttle-Mir/NASA-4	https://history.nasa.gov/SP-4225/nasa4/nasa4.htm
NASA-4: Failures to Communicate	Shuttle-Mir/NASA-4	https://history.nasa.gov/SP-4225/nasa4/nasa4.htm#communications
NASA history web article on Apollo 13	Apollo 13	https://history.nasa.gov/SP-350/ch-13-4.html
NASA mission archive on STS-9	STS-9	https://www.nasa.gov/mission_pages/shuttle/shuttlemissions/archives/sts-9.html
NASA mission archive on STS-27	STS-27	https://www.nasa.gov/mission_pages/shuttle/shuttlemissions/archives/sts-27.html
NASA mission archive on STS-61	STS-61	https://www.nasa.gov/mission_pages/shuttle/shuttlemissions/archives/sts-61.html
Timeline of notable ISS events	ISS	https://www.issnationallab.org/about/iss-timeline/
NASA mission archive on STS-72	STS-72	https://www.nasa.gov/mission_pages/shuttle/shuttlemissions/archives/sts-72.html
NASA mission archive on STS-74	STS-74	https://www.nasa.gov/mission_pages/shuttle/shuttlemissions/archives/sts-74.html
NASA mission archive on STS-75	STS-75	https://www.nasa.gov/mission_pages/shuttle/shuttlemissions/archives/sts-75.html
NASA mission archive on STS-114	STS-114	https://www.nasa.gov/mission_pages/shuttle/shuttlemissions/archives/sts-114.html
NASA mission archive on STS-2	STS-2	https://www.nasa.gov/mission_pages/shuttle/shuttlemissions/archives/sts-2.html
NASA mission archive on STS-82	STS-82	https://www.nasa.gov/mission_pages/shuttle/shuttlemissions/archives/sts-82.html
NASA mission archive on STS-86	STS-86	https://www.nasa.gov/mission_pages/shuttle/shuttlemissions/archives/sts-86.html
NASA STS-135 Press Kit	STS-135	https://www.nasa.gov/pdf/566071main_STS-135_Press_Kit.pdf
NASA web article on Apollo mission AS-204	Apollo 204 (Apollo 1)	https://nssdc.gsfc.nasa.gov/planetary/lunar/apollo1info.html
New York Times Article on STS-51L	Challenger STS-51L	https://www.nytimes.com/1986/01/29/us/shuttle-explosion-mission-control-silence-grief-fill-day-horror-long-dreaded.html
NPR web article on Challenger mission STS-51L	Challenger STS-51L	https://www.npr.org/templates/story/story.php?storyId=5175151
NPR web article on Challenger mission STS-51L	Challenger STS-51L	https://www.npr.org/templates/story/story.php?storyId=5174355
SP-4208 Living and Working in Space: A History of Skylab: Chapter 13 – Launching Skylab	Skylab station	https://history.nasa.gov/SP-4208/ch13.htm
SP-4208 Living and Working in Space: A History of Skylab: Chapter 14 – Saving Skylab	SL-2	https://history.nasa.gov/SP-4208/ch14.htm
The Flight of Apollo 13	Apollo 13	https://www.hq.nasa.gov/pao/History/apollo/apo13hist.html
The Gemini Program (1962-1966)	Project Gemini	https://nssdc.gsfc.nasa.gov/planetary/gemini.html
Web article on Columbia STS-107	Space Shuttle Columbia STS 107	http://www.americaspace.com/2015/02/01/lock-the-doors-columbias-final-flight-part-4/
Web article on Gemini VI	Gemini 6	http://www.spacefacts.de/mission/english/gemini-6.htm
Web article on ISS Expedition 54	ISS Expedition 54	http://www.spaceflightinsider.com/missions/iss/space-station-trio-returns-to-earth-after-record-setting-mission/
Web article on ISS spacewalk	ISS Expedition 54	http://www.spaceflightinsider.com/missions/iss/spacewalking-astronauts-finish-canadarm2-work-at-breakneck-speed/
Web article on Soyuz mission MS-07	Soyuz MS-07	http://www.spaceflightinsider.com/missions/iss/soyuz-ms-07-crew-back-on-earth-after-168-days-in-orbit/
Web article on STS-9, Spacelab	STS-9; Spacelab	http://www.spaceflightinsider.com/space-flight-history/spaceflight-heritage-sts-9-first-flight-spacelab/
